# Awareness and knowledge of Human Papillomavirus (HPV), HPV‐related cancers, and HPV vaccines in an uninsured adult clinic population

**DOI:** 10.1002/cam4.933

**Published:** 2016-10-17

**Authors:** Carmen Radecki Breitkopf, Lila J. Finney Rutten, Victoria Findley, Debra J. Jacobson, Patrick M. Wilson, Monica Albertie, Robert M. Jacobson, Gerardo Colón‐Otero

**Affiliations:** ^1^ Department of Health Sciences ResearchMayo ClinicRochesterMinnesota; ^2^ Mayo Clinic Robert D. and Patricia E. Kern Center for the Science of Health Care DeliveryRochesterMinnesota; ^3^ Volunteers in Medicine ClinicJacksonvilleFlorida; ^4^ Department of Health Sciences ResearchMayo ClinicJacksonvilleFlorida; ^5^ Department of Pediatric and Adolescent MedicineMayo ClinicRochesterMinnesota; ^6^ Hematology/OncologyMayo ClinicJacksonvilleFlorida

**Keywords:** Adult, health care disparities, knowledge, oncogenic viruses, papillomavirus vaccines

## Abstract

Human papillomavirus (HPV) vaccines offer primary prevention of cervical cancer and protection against other HPV‐associated cancers. HPV vaccine coverage in the United States (U.S.) remains low, particularly among older adolescents/young adults, and the uninsured. We assessed awareness and knowledge of HPV disease, HPV‐related cancers, and HPV vaccines among working, uninsured adults. Data from the 2014 Health Information National Trends Survey (HINTS 4, Cycle 4) were used as a benchmark. Patients were surveyed in late 2014 at the Volunteers in Medicine free clinic in Duval County, Florida. Surveys contained validated measures of HPV disease and vaccine knowledge; HPV‐related cancer knowledge was also assessed. Two‐hundred and ninety‐six surveys were analyzable with an 84% participation rate. Half (50.3%) of participants had heard of HPV, and 32.1% had heard of the HPV vaccine; in HINTS, these estimates were 63.6% and 62.7%, respectively (both *P* < 0.0001). In adjusted models, high HPV disease knowledge was associated with white race and increased education; high vaccine knowledge was associated with white race, increased education, and female sex. Recognition of HPV as a causative agent was 43.9% for cervical, 9.1% for anal, and 11.1% for throat cancers. For all HPV‐associated cancers, participants had lower knowledge/recognition relative to HINTS. The uninsured, socioeconomically disadvantaged adults we surveyed were unaware of a ubiquitous virus that can cause cancer and the existence of a vaccine to protect against it. These findings point to settings and populations in which initiatives to promote HPV vaccination as a cancer prevention tool remain critical.

## Introduction

Human papillomavirus (HPV) is a causative agent in cervical, anal, oropharyngeal, penile, and vaginal/vulvar cancers 1, 2, 3, 4, 5. HPV types 16 and 18 are responsible for >90% of HPV‐associated cancers; 6 however, a number of additional types have been identified as carcinogenic 7. Analysis of 2008–2012 data from the National Program of Cancer Registries (NPCR) and the Surveillance, Epidemiology, and End Results (SEER) Program, determined that approximately 30,700 incident cancers attributable to HPV occurred in the United States each year 8. The rates per 100,000 persons (age‐adjusted to the 2000 U.S. standard population) of histologically confirmed HPV‐associated cancers by site are 7.4 for cervical cancer (female only), 4.5 for oropharyngeal squamous cell carcinoma (SCC; 7.6 among males and 1.7 among females), 2.0 for vulvar SCC (female only), 1.5 for anal SCC (1.1 among males and 1.8 among females), and <1.0 for penile, vaginal, and rectal SCC 8.

Licensed, safe, and effective vaccines to protect against new HPV infections, including types 16 and 18, are currently available; these include bivalent, quadrivalent, and nonavalent vaccines, each initially licensed for administration according to a 3‐dose schedule. In 2014, the World Health Organization reviewed noninferiority data and evidence from studies with alternative dosing regimens 9, 10, 11, 12 and recommended a 2‐dose (0, 6 months “prime‐boost”) schedule of bivalent or quadrivalent vaccine for females 9–14 years of age, with vaccination of males receiving lower priority 13. Several countries have adopted 2‐dose regimens and gender‐restricted HPV vaccination programs, despite the clear public health importance of universal (both males and females) vaccination 14, 15. In the United States, a 2‐dose schedule is currently under consideration by the Advisory Committee on Immunization Practices (ACIP) but is not yet recommended. Presently, the ACIP recommends routine vaccination of both males and females at 11 or 12 years of age. Specific recommendations include administering HPV vaccine up to age 21 for males and up to age 26 for females, males who have sex with males, and immunocompromised individuals 16, 17, 18, 19. HPV vaccines offer primary prevention of cervical cancer and afford protection against other HPV‐associated cancers. In 2014, only 39.7% of girls and 21.6% of boys 13–17 years of age completed the HPV vaccine 3‐dose series in the United States 20. Rates of vaccination against HPV are even lower in the United States among 19–26 year olds, particularly among the uninsured 20. Populations vulnerable to health disparities, such as racial/ethnic minority groups, those with low income, limited English proficiency, or who are under‐ or uninsured, also have lower HPV vaccine completion rates 21. Lower rates of vaccination among these groups may compound already‐existing socioeconomic and racial/ethnic disparities for several HPV‐associated cancers – including cervical, oropharyngeal, and vaginal cancers 22.

Reasons for poor HPV vaccine coverage in the United States have been described and include parent‐related factors, with ‘lack of knowledge’ and lack of physician recommendation being primary reasons cited among parents for not vaccinating their children 20, 23. Additional barriers described by parents include inconsistent use of preventive services among adolescents and teens, the cost of vaccination, low perceived risk of HPV infection, and concerns around the potential impact on sexual behavior 23. Parental attitudes and financial concerns have been cited as barriers to offering the vaccine by healthcare providers 23. System‐level barriers, such as lack of school‐based vaccination program or mandates, and insurance coverage have also been frequently identified 23. These and other factors were thoroughly reviewed by Jacobson and colleagues 24.

The President's Cancer Panel report identified the need to increase HPV vaccination as a public health priority and outlined three goals toward achieving increased HPV vaccination coverage in the United States: reducing missed clinical opportunities to recommend and administer HPV vaccines; increasing acceptance of HPV vaccination; and maximizing access to HPV vaccination services 25. The research described herein was undertaken with these three goals in mind and a focus on traditionally underserved populations and populations suffering greater cancer burden. Specifically, the purpose of this study was to assess awareness and knowledge of HPV disease, HPV‐related cancers, and HPV vaccines among adults attending a community‐based volunteer clinic for the working uninsured. Little is known about whether individuals who receive their routine health care in volunteer‐based health care settings are reached by HPV vaccine messaging and cancer‐related education. Our research addresses this gap in the evidence base and characterizes HPV‐related knowledge and attitudes among an uninsured, working, and ethnically and racially diverse population.

## Materials and Methods

### Design and setting

This cross‐sectional, community‐based study was approved by the Institutional Review Board of Mayo Clinic and conducted at the Volunteers in Medicine Clinic (VIM) in Jacksonville, Florida (FL). The VIM clinic provides free primary care for working individuals without insurance who meet certain income requirements and live and/or work in Duval County, FL. Health care providers at VIM include volunteers who practice at other local health care organizations, in addition to full time staff. Approximately 1800 adult patients are seen annually; a majority (about 70%) of patients are female and over half are members of racial or ethnic minority groups. Currently, HPV vaccination is not offered at VIM due to cost considerations and a low volume of pediatric/adolescent patients.

### Instrument development and measures

The content of the survey was informed by general knowledge of the clinic population and review of existing measures of HPV vaccine attitudes and knowledge. Demographic characteristics included sex, age, race, ethnicity, marital status, employment status, number of children, and formal educational attainment. Awareness of HPV was assessed by a “Yes” or “No” response to the question: “Before today, had you ever heard of human papillomavirus (HPV)?” Those responding “yes” completed a 16‐item validated HPV knowledge measure assessing disease knowledge developed by Waller and colleagues 26. Example items include “HPV can be passed during sexual intercourse,” “A person could have HPV for many years without knowing it,” and “HPV can be cured with antibiotics.” Response options included “True,” “False,” and “Don't know,” with scoring allocating one point for each correct response, and zero points for incorrect or “Don't know” responses, summed across items, for a potential range of 0–16. Additionally, HPV‐related cancer knowledge was assessed in a series of nine items presented as follows: “HPV can cause ___ cancer” with five HPV‐related cancers (cervical, anal, throat, penile, vaginal/vulvar) and four cancers not currently known to be HPV‐related (lung, breast, prostate, skin) included; response options given were “True,” “False,” and “Don't know.” Respondents who had not heard of HPV were instructed to skip the disease and cancer knowledge items and move to the item measuring HPV vaccine awareness. HPV vaccine awareness was assessed by the question “Have you ever heard of a vaccine to protect against HPV (such as Gardasil or Cervarix)?” with “Yes” or “No” response options. Those responding “Yes” completed seven vaccine knowledge items from Waller et al. 26. Example items include “Girls who have had the HPV vaccine do not need a Pap smear/test when they are older,” “The HPV vaccines are most effective if given to people who have never had sex,” and “The HPV vaccines offer protection against all sexually transmitted infections.” The response options and scoring for these seven items was the same as described above for the disease knowledge items, with a potential range of 0–7.

Surveys were developed in English and translated into Spanish by a certified translator using translation and back‐translation techniques. The Spanish translation was subsequently reviewed locally for language appropriateness; this process resulted in minor modifications. The survey content and data collection methodology were pilot‐tested for a 1‐week period in VIM (March 20–25, 2014). Pilot‐testing demonstrated feasibility of recruitment (91% eligible patients were offered a survey by clinic staff), acceptability of a research survey in this setting (84% participation rate), and informed changes to the survey design and layout for ease of completion.

### Procedures

Volunteers in Medicine staff recruited a convenience sample of patients attending clinical appointments during the study period (September 10, 2014–October 24, 2014). Patients who were not in acute distress, understood English or Spanish, and provided oral consent to participate in a research study were offered a survey. Patients who returned a survey received a thank you gift and the HPV Fact Sheet developed by the Centers for Disease Control and Prevention.

Data from the 2014 Health Information National Trends Survey (HINTS 4, Cycle 4) regarding HPV knowledge and vaccine awareness were used as a national benchmark against which to compare data from the VIM sample. HINTS is a nationally representative survey of the U.S. adult population focused on cancer‐related knowledge, attitudes, and behavior 27. Data for HINTS 4, Cycle 4, were collected between July and November 2014 through self‐administered mailed questionnaires. Further details regarding HINTS methodology and questionnaires can be found on the HINTS website 28.

### Analysis

Demographic characteristics were assessed overall and for those who had heard of HPV and the HPV vaccine. All three knowledge scores were created by equal weight summation. Furthermore, if survey participants responded as being unaware of HPV or the HPV vaccine, their respective knowledge scores were imputed to zero. High knowledge for HPV disease, HPV vaccines, and HPV‐related cancer was defined as having a knowledge score in the upper quartile of positive values (≥75th percentile of knowledge scores ≥1). Odds ratios (OR) and 95% confidence intervals were estimated using logistic regression to assess the association of demographic characteristics with having high knowledge (as defined above) of HPV disease, HPV‐related cancers, and the HPV vaccine, respectively. Additionally, estimates of association were derived for each individual cancer comprising the HPV‐related cancer knowledge score. Proportions on the benchmark items were calculated and tested using a one‐sample proportion test. The null hypothesis being that the VIM proportion equals the national benchmark (HINTS) estimate. HINTS data were weighted to provide estimates of the U.S. population as well as correct for nonresponse bias. All analyses were performed using SAS 9.4. Statistical comparisons were two‐sided and were considered significant at the *P* < 0.05 level.

## Results

During the study period, a total of 356 patients were eligible to participate. Of these, 301 patients accepted a survey and 55 declined (84% participation rate). Of the 301 surveys returned, five were excluded from analysis due to excessive missing data; a total of 296 surveys were included in the analyses. While the study participants were all adults, 230 (79%) indicated that they had children. Of 183 participants who indicated their children's age, 111 (61%) had at least one child who would be age‐eligible for HPV vaccination (ages: 9–26).

The study sample closely reflected the overall demographics of the VIM patient population (described above). Specifically, 78% of the sample was female, 51% were black or African American and 10% were Hispanic, with a median age of 49 years. Additional demographic characteristics are shown in Table**** 1. The sample differed somewhat from the population of Duval County, FL, which is 51.5% female, 29.4% black or African American, and 8.1% Hispanic, with a median age of 35.9 years (http://factfinder.census.gov/faces/tableservices/jsf/pages/productview.xhtml?src=CF).

**Table 1:** Demographics for total sample and by HPV disease awareness and HPV vaccine awareness

Characteristic	Total sample (*n* = 296)	Heard of HPV (Yes) (*n* = 149)	Heard of HPV Vaccine (Yes) (*n* = 95)
Age (years)			
Mean (SD)	47.1 (10.1)	45.2 (10.5)	45.4 (10.0)
Median (Q1, Q3)	49 (41, 54)	46 (38, 53)	47.5 (39, 52)
	*N*, %^a^	*N*, %^a^	*N*, %^a^
Sex			
Female	225 (77.9)	129 (87.2)	85 (90.4)
Male	64 (22.1)	19 (12.8)	9 (9.6)
Race			
Black	150 (50.7)	73 (49.0)	40 (42.1)
White	114 (38.5)	65 (43.6)	51 (53.7)
Other	32 (10.8)	11 (7.4)	4 (4.2)
Ethnicity			
Hispanic	25 (9.8)	11 (8.0)	10 (11.2)
Not Hispanic	231 (90.2)	127 (92.0)	79 (88.8)
Education			
≤High school	140 (49.0)	51 (34.9)	27 (29.3)
Some college/technical school	102 (35.7)	68 (46.6)	45 (48.9)
≥College	44 (15.4)	27 (18.5)	20 (21.7)
Marital status			
Single	71 (24.5)	36 (24.3)	23 (24.5)
Married/partnered	110 (37.9)	57 (38.5)	37 (39.4)
Other^b^	109 (37.6)	55 (37.2)	34 (36.2)
Have children			
Yes	230 (78.8)	110 (73.8)	73 (76.8)
No	62 (21.2)	39 (26.2)	22 (23.2)
Number of children			
Mean (SD)	2.12 (1.78)	1.77 (1.54)	1.89 (1.51)
Median (Q1, Q3)	2 (1, 3)	2 (0, 3)	2 (1, 3)

HPV, Human papillomavirus; SD, standard deviation.

a Frequencies and percent of nonmissing responses are reported.

b Separated, divorced, widowed.

Overall, 50.3% (*n* = 149) of the sample reported they had heard of human papillomavirus or HPV, and 32.1% (*n* = 95) had heard of a vaccine to protect against HPV (such as Gardasil or Cervarix) (Table**** 1). These rates are lower than estimates of awareness of HPV (63.6%) and the HPV vaccine (62.7%) obtained in HINTS data (both *P* < 0.0001).

HPV disease, HPV vaccine, and HPV cancer‐related knowledge questions ranged from 0 to 16 items, 0–7 items, and 0–7 items, respectively. For patients who were aware of HPV or the HPV vaccine, the median (Q1, Q3) correct was 7 (2, 10), 3 (0, 4), and 1 (0, 4) for HPV disease, HPV vaccine, and HPV cancer knowledge, respectively. In adjusted models, whites and those with more education were more likely to have high HPV disease knowledge. Similarly, whites, females, and those with more education were more likely to have high HPV vaccine knowledge (Table**** 2). In adjusted models, there were no significant associations between demographic characteristics and high total scores of HPV‐related cancer knowledge; however, the strongest association was with increased education (*P* = 0.067).

**Table 2:** Logistic regression assessing demographic predictors of high knowledge^a^

Characteristic	HPV knowledge		HPV vaccine knowledge		Cancer knowledge	
	OR	95% CI	OR	95% CI	OR	95% CI
Unadjusted models						
Age (continuous)	0.96	(0.93, 0.99)	0.98	(0.96, 1.01)	0.98	(0.95, 1.02)
Sex (Female)	2.22	(0.90, 5.48)	**2.83**	(1.16, 6.94)	1.21	(0.47, 3.09)
Race (white)	**2.15**	(1.15, 4.01)	**1.88**	(1.05, 3.36)	2.10	(0.99, 4.45)
Ethnicity (Hispanic)	1.16	(0.41, 3.27)	0.98	(0.35, 2.74)	1.08	(0.30, 3.84)
Education (> High School)	**2.97**	(1.49, 5.90)	**4.60**	(2.31, 9.16)	**2.93**	(1.26, 6.82)
Adjusted models						
Age (continuous)	0.95	(0.92, 0.99)	0.98	(0.95, 1.02)	0.98	(0.94, 1.01)
Sex (Female)	1.99	(0.77, 5.17)	**2.93**	(1.06, 8.08)	1.24	(0.44, 3.50)
Race (White)	**2.58**	(1.28, 5.21)	**2.39**	(1.21, 4.68)	1.63	(0.72, 3.68)
Ethnicity (Hispanic)	1.03	(0.35, 3.05)	0.85	(0.28, 2.54)	1.02	(0.28, 3.75)
Education (> High School)	**2.34**	(1.14, 4.84)	**5.04**	(2.30, 11.06)	2.25	(0.94, 5.38)

CI, confidence interval; OR, odds ratio; HPV, Human Papillomavirus; Significant ORs in boldface.

a High knowledge defined as a knowledge score ≥75th percentile (of knowledge scores >1). Referent groups: Sex (Male); Race (nonwhite); Ethnicity (non‐Hispanic); and Education (< High School).

With regard to the knowledge items addressing specific cancers, knowledge was greatest for cervical cancer, with 43.9% (*n* = 130) answering correctly that HPV can cause cervical cancer. Importantly, only 9.1% (*n* = 27) of respondents marked the correct answer (“True”) for “HPV can cause anal cancer.” When benchmarked against national estimates obtained in HINTS 2014 data, VIM survey respondents were less knowledgeable of HPV as a cancer‐causing agent for each of the cancers that were included in both surveys (Table**** 3). Furthermore, only 26% (*n* = 77) of VIM survey respondents gave the correct answer of “False” to the item “HPV can cause lung cancer” and less than one‐quarter correctly answered “False” to the item “HPV can cause breast cancer.” Associations between demographic characteristics and correct knowledge of individual cancers revealed only sex/gender associations for cervical (OR = 2.44; 95% CI = [1.33, 4.55]) and vulvar/vaginal cancer (OR = 2.33; 95% CI = [1.16, 4.55]) such that females were more likely to answer these items correctly. Similarly, nonwhites were less likely to have correct knowledge of lung and skin cancers. Also, those with more than a high school education were more likely to have correct knowledge of cervical, vulvar, prostate, anal, lung, and skin cancers (results not shown).

**Table 3:** HPV‐related cancer knowledge among VIM sample and HINTS Cycle4 respondents

HPV can cause	VIM sample*N* (%) correct	HINTS Cycle 4^a^ *N* (%) correct	*P*‐value
Cervical cancer^b^	130 (43.9)	1649 (49.0)	0.0803
Penile cancer^b^	34 (11.5)	574 (18.1)	0.0031
Anal cancer^b^	27 (9.1)	526 (16.0)	0.0012
Throat cancer^b^	33 (11.1)	638 (18.5)	0.0011
Vaginal/vulvar cancer^b^	91 (30.7)	–	–
Lung cancer^c^	77 (26.0)	–	–
Breast cancer^c^	70 (23.6)	–	–
Prostate cancer^c^	33 (11.1)	–	–
Skin cancer^c^	57 (19.3)	–	–

VIM, volunteers in medicine clinic; HPV, Human Papillomavirus; –, data not available.

a Health Information National Trends Survey (HINTS) data are unweighted responses and estimated weighted percent.

b Correct answer = yes.

c Correct answer = no.

## Discussion

Awareness and knowledge of HPV disease, HPV vaccines, and HPV‐related cancers was low among working, uninsured adult patients receiving care in a volunteer‐based community health clinic. Only half of survey respondents had heard of HPV; this estimate is similar to that reported by Fowler and colleagues among a community‐based sample of Latinas in the United States 29. Further, less than one‐third of VIM clinic patients surveyed had heard of a vaccine to prevent HPV and a majority of our respondents did not know the causative relationship between HPV and many cancers. The low rates of knowledge of HPV disease and the availability of prophylactic vaccines observed in this sample of medically underserved adults in the United States are consistent with rates reported among a population‐based survey of young adult women in Morocco 30 and a systematic review of 14 studies conducted in African countries 31. Knowledge of the causal link between cancer and HPV was highest for cervical cancer. However, fewer than half of respondents were aware of this association. Knowledge of the link between HPV and throat cancer was strikingly low, at 11% of the sample. This finding is consistent with a reported range of between 1 and 44% for general (nonmedical or dental professional) population samples in a recent systematic review 32. Oropharyngeal cancer affects both men and women and is increasing in incidence in several developed countries, including the United States 33, 34, 35. The lack of knowledge of HPV as a risk factor for oropharyngeal cancer, the increasing prevalence of oropharyngeal cancer, an increased risk of this cancer for males as compared to females, and unavailability of screening 35 should draw attention to the need for vaccine efficacy data in this cancer and universal HPV vaccine recommendations in countries where they currently do not exist.

These findings underscore a crucial need for strategies to educate medically underserved adults about HPV and to offer information about the HPV vaccine and, if resources permit, offer the vaccine in all clinic settings. Volunteer clinics should be a priority for HPV vaccine initiatives because they reach particular populations (e.g., uninsured, female, racial minority) that have disproportionately lower HPV vaccine coverage and higher rates of several HPV‐associated cancers 22. While this clinic assists working individuals without insurance who meet certain income requirements, we estimate that 40–50% would have at least one child who is eligible for the HPV vaccine. Increasing the knowledge of the clinic patients about the HPV vaccine and community resources available for children to be vaccinated may mediate an increase in vaccinations of their age‐eligible children. Maximizing access to HPV information, factual knowledge, and vaccination, including catch‐up vaccination for adults up to age 26, is consistent with the recommendations of the American College of Obstetricians and Gynecologists, the American College of Physicians, the Advisory Committee on Immunization Practices and addresses all three of the goals within the President's Cancer Panel Report 25. Indeed, this report recommends increasing the range of settings in which HPV vaccines may be administered.

Decades of work in cancer prevention, early detection, and treatment have resulted in declining death rates for many cancers in the United States; however, the gains afforded by such efforts have not been equally enjoyed by those belonging to economically and socially disadvantaged groups 36. Cancer remains a highly‐feared disease and a complex condition that is associated with significant morbidity and mortality. Communication strategies that frame HPV vaccines as vaccines to prevent cancer may increase acceptance and coverage. Toward this end, the nation's National Cancer Institute‐designated cancer centers recently endorsed a statement promoting HPV vaccination as a cancer prevention tool, in the first unanimous endorsement of its kind (http://www.asco.org/sites/www.asco.org/files/nci_hpv_consensus_statement_012716.pdf).

Prior research suggests that educational efforts emphasizing the diseases that vaccines prevent may be more effective in reducing vaccine hesitancy than educational interventions aimed at dispelling misconceptions about the vaccine 37. Thus, adopting a cancer‐prevention dialog may effectively divert the provider‐adolescent‐parent conversation away from sexual activity. Instead, the conversation could be directed toward a discussion of primary and secondary prevention of future cancers by getting the HPV vaccine and not initiating (or stopping) cancer‐risk behaviors that often start in young adulthood (e.g., tobacco use, tanning).

This study reveals gaps in knowledge and awareness of HPV vaccines in a United States sample of uninsured, socioeconomically disadvantaged adults when benchmarked against national estimates obtained during the same year. These gaps in knowledge potentially exist in other, similarly disadvantaged groups in the United States more than 10 years after the vaccine was licensed for use among females, and several years after being recommended for males. Our findings suggest that some of the most vulnerable individuals remain unaware of a ubiquitous virus that can cause cancer and the existence of a safe and effective vaccine to protect against it. These findings, while not without the limitations associated with a convenience sample of relatively small size obtained from a single clinic setting, are important and point to settings and populations in which HPV vaccine initiatives remain critical.

## Conflict of Interest

None declared.

## References

[1] Walboomers, J. M. , M. V. Jacobs , M. M. Manos , et al. 1999 Human papillomavirus is a necessary cause of invasive cervical cancer worldwide. J. Pathol. 189:12–19. 10451482 10.1002/(SICI)1096-9896(199909)189:1<12::AID-PATH431>3.0.CO;2-F

[2] Muñoz, N. , F. X. Bosch , S. de Sanjosé , et al. 2003 Epidemiologic classification of human papillomavirus types associated with cervical cancer. N. Engl. J. Med. 348:518–527. 12571259 10.1056/NEJMoa021641

[3] IARC Working Group on the Evaluation of Carcinogenic . 2012 Risks to Humans. Biological agents: a review of human carcinogens. IARC Monogr. Eval. Carcinog. Risks Hum. 100B:1–441.

[4] Forman, D. , C. de Martel , C. J. Lacey , et al. 2012 Global burden of human papillomavirus and related diseases. Vaccine 30(Suppl 5):F12–F23. 23199955 10.1016/j.vaccine.2012.07.055

[5] zur Hausen, H. . 2009 Papillomaviruses in the causation of human cancers ‐ a brief historical account. Virology 384:260–265. 19135222 10.1016/j.virol.2008.11.046

[6] Lowy, D. R. , and J. T. Schiller . 2012 Reducing HPV‐associated cancer globally. Cancer Prev. Res. (Phila.) 5:18–23. 22219162 10.1158/1940-6207.CAPR-11-0542 PMC3285475

[7] Bouvard, V. , R. Baan , K. Straif , et al. 2009 A review of human carcinogens–Part B: biological agents. Lancet Oncol. 10:321–322. 19350698 10.1016/s1470-2045(09)70096-8

[8] Viens, L. J. , S. J. Henley , M. Watson , et al. 2016 Human papillomavirus‐associated cancers ‐ United States, 2008‐2012. MMWR Morb. Mortal. Wkly Rep. 65:661–666. 27387669 10.15585/mmwr.mm6526a1

[9] Dobson, S. R. , S. McNeil , M. Dionne , et al. 2013 Immunogenicity of 2 doses of HPV vaccine in younger adolescents vs 3 doses in young women: a randomized clinical trial. JAMA 309:1793–1802. 23632723 10.1001/jama.2013.1625

[10] Kreimer, A. R. , M. E. Sherman , V. V. Sahasrabuddhe , and M. Safaeian . 2015 The case for conducting a randomized clinical trial to assess the efficacy of a single dose of prophylactic HPV vaccines among adolescents. J. Natl Cancer Inst. 107: dju436. 25650316 10.1093/jnci/dju436 PMC4326307

[11] Lamontagne, D. S. , V. D. Thiem , V. M. Huong , Y. Tang , and K. M. Neuzil . 2013 Immunogenicity of quadrivalent HPV vaccine among girls 11 to 13 years of age vaccinated using alternative dosing schedules: results 29 to 32 months after third dose. J. Infect. Dis. 208:1325–1334. 23901077 10.1093/infdis/jit363

[12] Sankaranarayanan, R. , P. R. Prabhu , M. Pawlita , et al. 2016 Immunogenicity and HPV infection after one, two, and three doses of quadrivalent HPV vaccine in girls in India: a multicentre prospective cohort study. Lancet Oncol. 17:67–77. 26652797 10.1016/S1470-2045(15)00414-3 PMC5357737

[13] World Health Organization . 2014 Human papillomavirus vaccines: WHO position paper, October 2014. Wkly Epidemiol. Rec. 89:465–491. 25346960

[14] Audisio, R. A. , G. Icardi , A. M. Isidori , et al. 2016 Public health value of universal HPV vaccination. Crit. Rev. Oncol. Hematol. 97:157–167. 26346895 10.1016/j.critrevonc.2015.07.015

[15] Crosignani, P. , A. de Stefani , G. M. Fara , et al. 2013 Towards the eradication of HPV infection through universal specific vaccination. BMC Public Health 13:642. 23845195 10.1186/1471-2458-13-642 PMC3751659

[16] Stokley, S. , J. Jeyarajah , D. Yankey , et al. 2014 Human papillomavirus vaccination coverage among adolescents, 2007‐2013, and postlicensure vaccine safety monitoring, 2006‐2014–United States. MMWR Morb. Mortal. Wkly Rep. 63:620–624. 25055185 PMC5779422

[17] Markowitz, L. E. , E. F. Dunne , M. Saraiya , et al. 2014 Human papillomavirus vaccination: recommendations of the advisory committee on immunization practices (ACIP). MMWR Recomm. Rep. 63(RR05):1–30. 25167164

[18] Petrosky, E. , J. A. Jr Bocchini , S. Hariri , et al. 2015 Use of 9‐valent human papillomavirus (HPV) vaccine: updated HPV vaccination recommendations of the advisory committee on immunization practices. MMWR Morb. Mortal. Wkly Rep. 64:300–304. 25811679 PMC4584883

[19] Committee Opinion No . 2015 641: human papillomavirus vaccination. Obstet. Gynecol. 126:e38–e43. 26287792 10.1097/AOG.0000000000001052

[20] Reagan‐Steiner, S. , D. Yankey , J. Jeyarajah , et al. 2015 National, regional, state, and selected local area vaccination coverage among adolescents aged 13–17 years–United States, 2014. MMWR Morb. Mortal. Wkly Rep. 64:784–792. 26225476 10.15585/mmwr.mm6429a3 PMC4584833

[21] Jeudin, P. , E. Liveright , M. G. Del Carmen , and R. B. Perkins . 2014 Race, ethnicity, and income factors impacting human papillomavirus vaccination rates. Clin. Ther. 36:24–37. 24417783 10.1016/j.clinthera.2013.11.001

[22] Jemal, A. , E. P. Simard , C. Dorell , et al. 2013 Annual Report to the Nation on the Status of Cancer, 1975‐2009, featuring the burden and trends in human papillomavirus(HPV)‐associated cancers and HPV vaccination coverage levels. J. Natl Cancer Inst. 105:175–201. 23297039 10.1093/jnci/djs491 PMC3565628

[23] Holman, D. M. , V. Benard , K. B. Roland , M. Watson , N. Liddon , and S. Stokley . 2014 Barriers to human papillomavirus vaccination among US adolescents: a systematic review of the literature. JAMA Pediatr. 168:76–82. 24276343 10.1001/jamapediatrics.2013.2752 PMC4538997

[24] Jacobson, R. M. , and A. A. Agunwamba , J. L. St Sauver , L. J. Finney Rutten . 2016 The most effective and promising population health strategies to advance human papillomavirus vaccination. Expert. Rev. Vaccines 15:257–269. 26559567 10.1586/14760584.2016.1116947 PMC6684098

[25] Accelerating HPV vaccine uptake: urgency for action to prevent cancer . 2014 A Report to the President of the United States from the President's Cancer Panel. Bethesda, MD: National Cancer Institute. Available from URL: http://deainfo.nci.nih.gov/advisory/pcp/annualReports/HPV/index.htm (accessed May, 9, 2016).

[26] Waller, J. , R. Ostini , L. A. Marlow , K. McCaffery , and G. Zimet . 2013 Validation of a measure of knowledge about human papillomavirus (HPV) using item response theory and classical test theory. Prev. Med. 56:35–40. 23142106 10.1016/j.ypmed.2012.10.028

[27] Nelson, D. E. , G. L. Kreps , and B. W. Hesse , et al. 2004 The health information National Trends Survey (HINTS): development, design, and dissemination. J. Health Commun.. 9:443–460; discussion 81‐84. 15513791 10.1080/10810730490504233

[28] Health Information National Trends Survey . 2016 Cycle 4, English version. Annotated form and Methodology Report. Available from URL: http://hints.cancer.gov [accessed 9 May 2016].

[29] Fowler, B. , J. Bodson , E. L. Warner , J. Dyer , and D. Kepka . 2016 Poor HPV vaccine‐related awareness and knowledge among Utah Latinas overdue for recommended cancer screenings J. Community Health. 41:825–837. 26860277 10.1007/s10900-016-0160-3

[30] Zouheir, Y. , S. Daouam , S. Hamdi , A. Alaoui , and T. Fechtali . 2016 Knowledge of human papillomavirus and acceptability to vaccinate in adolescents and young adults of the Moroccan population. J. Pediatr. Adolesc. Gynecol. 29:292–298. 26612116 10.1016/j.jpag.2015.11.002

[31] Cunningham, M. S. , C. Davison , and K. J. Aronson . 2014 HPV vaccine acceptability in Africa: a systematic review. Prev. Med. 69:274–279. 25451327 10.1016/j.ypmed.2014.08.035

[32] Dodd, R. H. , J. Waller , and L. A. Marlow . 2016 Human papillomavirus and head and neck cancer: psychosocial impact in patients and knowledge of the link ‐ a systematic review. Clin. Oncol. (R. Coll. Radiol.) 28:421–439. 26996812 10.1016/j.clon.2016.02.012 PMC4914608

[33] Gooi, Z. , J. Y. Chan , and C. Fakhry . 2016 The epidemiology of the human papillomavirus related to oropharyngeal head and neck cancer. Laryngoscope 126:894–900. 26845348 10.1002/lary.25767

[34] Chaturvedi, A. K. , E. A. Engels , W. F. Anderson , and M. L. Gillison . 2008 Incidence trends for human papillomavirus‐related and ‐unrelated oral squamous cell carcinomas in the United States. J. Clin. Oncol. 26:612–619. 18235120 10.1200/JCO.2007.14.1713

[35] Chaturvedi, A. K. , E. A. Engels , R. M. Pfeiffer , et al. 2011 Human papillomavirus and rising oropharyngeal cancer incidence in the United States. J. Clin. Oncol. 29:4294–4301. 21969503 10.1200/JCO.2011.36.4596 PMC3221528

[36] Siegel, R. L. , K. D. Miller , and A. Jemal . 2015 Cancer statistics, 2015. CA Cancer J. Clin. 65:5–29. 25559415 10.3322/caac.21254

[37] Horne, Z. , D. Powell , J. E. Hummel , and K. J. Holyoak . 2015 Countering antivaccination attitudes. Proc. Natl Acad. Sci. USA 112:10321–10324. 26240325 10.1073/pnas.1504019112 PMC4547299

